# Characterization of *FLOWERING LOCUS C* Homologs in Apple as a Model for Fruit Trees

**DOI:** 10.3390/ijms21124562

**Published:** 2020-06-26

**Authors:** Hidenao Kagaya, Naoko Ito, Tomoki Shibuya, Sadao Komori, Kazuhisa Kato, Yoshinori Kanayama

**Affiliations:** 1 Graduate School of Agricultural Science, Tohoku University, Aoba-ku, Sendai 980-8572, Japan; ba7.2025@gmail.com (H.K.); naoko.ito.p6@dc.tohoku.ac.jp (N.I.); 2 Faculty of Life and Environmental Science, Shimane University, Matsue 690-8504, Japan; tomoki.s.t.f@gmail.com; 3 Faculty of Agriculture, Iwate University, Morioka 020-8550, Japan; komoris@iwate-u.ac.jp

**Keywords:** *Malus domestica*, Rosaceae, juvenility, FLOWERING LOCUS C, flowering

## Abstract

To elucidate the molecular mechanism of juvenility and annual flowering of fruit trees, *FLOWERING LOCUS C* (*FLC*), an integrator of flowering signals, was investigated in apple as a model. We performed sequence and expression analyses and transgenic experiments related to juvenility with annual flowering to characterize the apple *FLC* homologs *MdFLC*. The phylogenetic tree analysis, which included other MADS-box genes, showed that both MdFLC1 and MdFLC3 belong to the same FLC group. MdFLC1c from one of the *MdFLC1* splice variants and MdFLC3 contain the four conserved motives of an MIKC-type MADS protein. The mRNA of variants *MdFLC1a* and *MdFLC1b* contain intron sequences, and their deduced amino acid sequences lack K- and C-domains. The expression levels of *MdFLC1a*, *MdFLC1b*, and *MdFLC1c* decreased during the flowering induction period in a seasonal expression pattern in the adult trees, whereas the expression level of *MdFLC3* did not decrease during that period. This suggests that *MdFLC1* is involved in flowering induction in the annual growth cycle of adult trees. In apple seedlings, because phase change can be observed in individuals, seedlings can be used for analysis of expression during phase transition. The expression levels of *MdFLC1b*, *MdFLC1c*, and *MdFLC3* were high during the juvenile phase and low during the transitional and adult phases. Because the expression pattern of *MdFLC3* suggests that it plays a specific role in juvenility, *MdFLC3* was subjected to functional analysis by transformation of *Arabidopsis*. The results revealed the function of *MdFLC3* as a floral repressor. In addition, *MdFT* had CArG box-like sequences, putative targets for the suppression of flowering by MdFLC binding, in the introns and promoter regions. These results indicate that apple homologs of FLC, which might play a role upstream of the flowering signals, could be involved in juvenility as well as in annual flowering. Apples with sufficient genome-related information are useful as a model for studying phenomena unique to woody plants such as juvenility and annual flowering.

## 1. Introduction

Fruit trees, which are perennial woody plants, have a long juvenile period after germination and before flowering and fruit set. Because fruit quality cannot be evaluated during this juvenile phase, it is necessary to address the long juvenile period of fruit trees with regard to fruit tree breeding. Lengths of juvenile periods differ among species, and apple requires six to eight years of juvenility [1]. Although some cultivation techniques to shorten the juvenile period have been proposed, such as plant hormone treatment, grafting to dwarf rootstocks, suppression of dormancy, and adjustment of cultivation conditions [2,3,4], little is known about the molecular mechanisms of juvenility. It is more difficult to elucidate the mechanism underlying the control of juvenility in fruit trees than in herbaceous plants because fruit trees take longer to grow and are larger in size. Apple is one of the earliest plants whose genome sequences have been reported as woody plants and fruit tree [5], and is utilized as a model because of its extensive genome information [6,7]. Therefore, apples are useful for studying phenomena unique to woody plants such as juvenility and annual flowering.

In general, juvenility is stronger in young seedlings and gradually weakens as age progresses. There is also known to be a gradient of juvenility in individual trees; that is, juvenility is stronger at the base of trunks and branches and becomes weaker approaching the tip [8]. Genes that are potentially related to juvenility have been reported in studies of homologs of flowering-related genes from *Arabidopsis*, which is a model herbal plant. Previous studies focused on the relationship between juvenility and the apple homologs of *TERMINAL FLOWER 1* (*TFL1*), *FLOWERING LOCUS T* (*FT*), *LEAFY* (*LFY*), and *APETALA1* (*AP1*), which are floral meristem identity-related genes in *Arabidopsis*. The expression of apple *TFL1* homolog *MdTFL* is high during the juvenile phase, and the expression of apple *FT* homolog *MdFT* is high during the adult phase [9]. The overexpression of apple homologs of *LFY* and *AP1* in *Arabidopsis* promotes flowering, and the overexpression of *MdTFL* delays flowering [10,11,12]. In citrus, the overexpression of *LFY* or *AP1* of *Arabidopsis* reportedly decreases the juvenile period from seven years to two years [13], and the methylation of the *LEAFY* homolog is thought to be involved in juvenility [14]. However, most of these genes are located relatively downstream of the flowering pathway, and in order to clarify the molecular mechanism of juvenility, it is necessary to analyze genes farther upstream.

There are several flowering pathways, including the photoperiod, vernalization, gibberellin, and autonomous pathways [15]. The photoperiod and vernalization-dependent pathways are controlled by environmental factors, and the gibberellin-dependent pathway is comprised of a group of genes related to the synthesis and signal transduction of gibberellin. In contrast, the autonomous pathway is dependent on endogenous growth-related factors. Juvenility is not reduced by environmental factors such as temperature and day length, but is reduced by growth over several years, which suggests that juvenility is controlled by endogenous growth-related factors. Some genes in the autonomous pathway are known to induce flowering by suppressing the expression of *FLOWERING LOCUS C* (*FLC*) [16]. *FLC* plays a role as a key regulator of the autonomous and vernalization pathways and inhibits flowering by suppressing the expression of floral induction genes *SOC1* and *FT* in *Arabidopsis* [17,18,19]. Since *FLC* is a key gene for the flowering pathways, including the autonomous pathway, it can be expected to play a role in the suppression of flowering in the juvenile phase.

There have been some reports on *FLC* homologs in fruit trees. *FLC* homologs have been identified in apple, and divergent functions have been suggested based on their nucleotide sequences [20]. Two of these exhibit increased expression during dormancy and decreased expression with dormancy release, which suggests that they repress flowering as described in *Arabidopsis*. In fact, the possibility that the *FLC*-like gene is a candidate gene is mentioned in the genome-wide association mapping of the flowering period in apple [21]. In other apple *FLC*-like genes, the repression of bud outgrowth during dormancy and the promotion of flowering under non-chilling conditions have been reported [22,23]. Among other Rosaceae fruit trees, the *FLC* homolog in peach (*Prunus persica*) has not been reported to be associated with dormancy, and its role has not been elucidated [24]. No *FLC* homolog has been found in Japanese apricot (*Prunus mume*) either [25]. In fruit trees other than Rosaceae, changes in the expression and splicing of an *FLC*-like gene were reported in trifoliate orange (*Poncirus trifoliata*) [26]. Although these reports provide some information about the role of *FLC* in fruit trees, information about the relationship between juvenility and *FLC* homologs is still lacking. Therefore, to investigate the physiological roles of apple *FLC* homolog *MdFLC* at the molecular level, sequence and expression analyses and transgenic experiments were performed.

## 2. Results

### 2.1. cDNA Isolation and Phylogenetic Tree Analysis of MdFLC

Nine apple expressed sequence tag (EST) sequences were obtained by a BLAST search in DNA Data Bank of Japan (DDBJ, http://blast.ddbj.nig.ac.jp) using the amino acid sequence of the MADS region of *Arabidopsis FLC*. The contig sequences corresponding to these EST sequences were searched in GDR (https://www.rosaceae.org), a Rosaceae genome database, to obtain four contig sequences. Six EST or contig sequences remained after excluding duplicate sequences. A phylogenetic tree was prepared based on the amino acid sequences of these six genes and MADS-box genes from *Arabidopsis* and apple, and three of the six sequences were assigned to the same group as *Arabidopsis FLC*. Next, one of the three sequences was detected by PCR using cDNA from the juvenile phase of apple seedlings with primers specific to the three sequences and designated as *MdFLC1* (MD05G1037100) [23]. Three kinds of mRNA sequence were obtained by the RACE method and designated as splice variants *MdFLC1a* (accession number LC550081), *MdFLC1b* (LC550082), and *MdFLC1c* (LC550083).

PCR was performed using cDNA from the juvenile phase of apple seedlings with degenerate primers in the MADS region, and 10 kinds of MADS box-like sequences were obtained. One sequence among them was found to be juvenile phase-specific and homologous to *Arabidopsis FLC*; it was designated as *MdFLC3* (MD10G1041100) [23].

**Figure 1:**
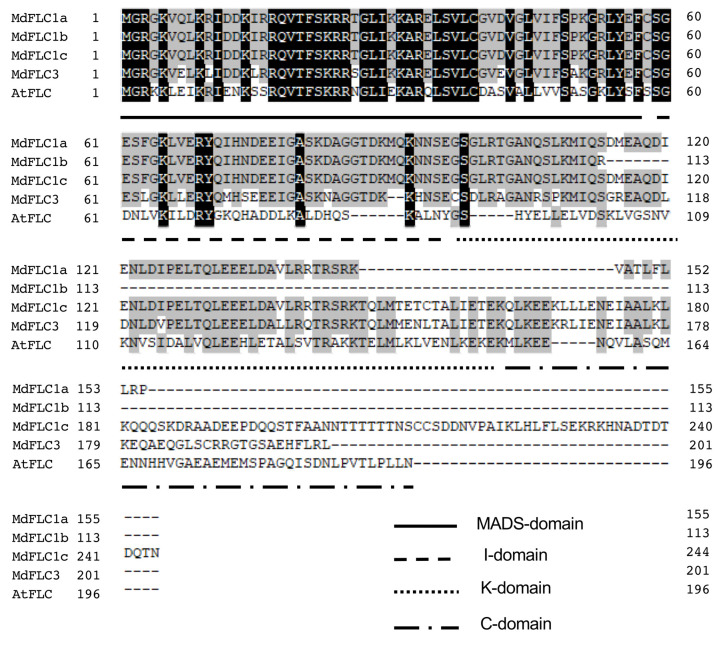
Amino acid sequence alignment of MdFLC and AtFLC. The MADS-, K-, I-, and C-domains are underlined. Identical amino acids for five and less proteins are shown in black and gray boxes, respectively.

**Figure 2:**
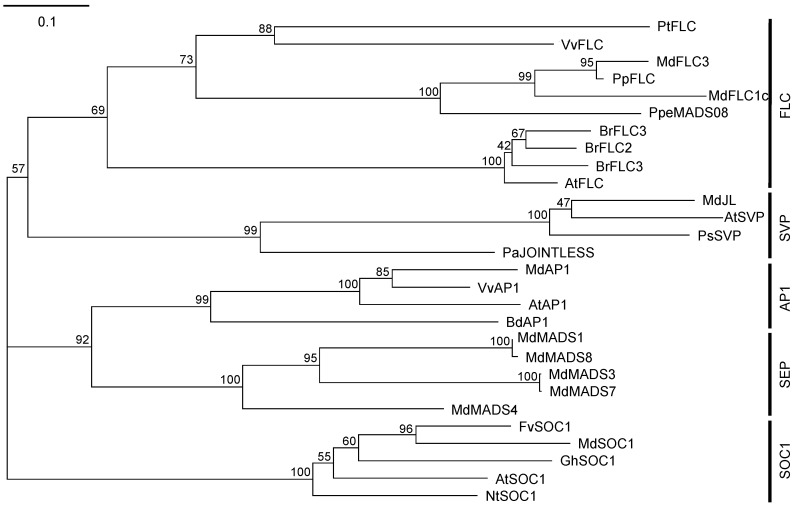
A phylogenetic tree based on the amino acid sequences of FLC, SVP, AP1, SEP, and SOC1 homologs from various species. The tree was constructed by the neighbor-joining method after sequence alignment using the ClustalW program. Branch numbers refer to percentage of replicates that support the branch using the bootstrap method (1000 replicates). The scale bar corresponds to 0.1 amino acid substitutions per residue. The accession numbers of the proteins added to construct the phylogenetic tree are as follows: PtFLC (EU497676), VvFLC (GU133630), PpFLC (KP164015), BrFLC1 (DQ866874), BrFLC2, (DQ866875), BrFLC3 (DQ866876), MdJOINTLESS (DQ402055), AtSVP (AF211171), PsSVP (AY830919), PaJOINTLESS (EU332978), VvAP1 (GU133634), MdAP1 (EU672877), AtAP1 (BT004113), BdAP1 (HQ588324), MdMADS1 (U78947), MdMADS3 (U78949), MdMADS4 (U78950), MdMADS7 (AJ000760), MdMADS8 (AJ001681), FvSOC1 (FJ531999), MdSOC1 (DQ846833), GhSOC1 (JF701982), NtSOC1 (JQ686938), AtSOC1 (AY093967).

Figure 1 shows the amino acid sequence alignment of MdFLC1, MdFLC3, and AtFLC (AF537203) from *Arabidopsis*. MdFLC1c, which was the longest of the *MdFLC1* mRNA variants, and MdFLC3 contained MADS-, K-, I-, and C-domains with AtFLC. A phylogenetic tree of the amino acid sequence of MdFLC1c, MdFLC3, and FLC homologous proteins from other plants was prepared with other MADS-box proteins based on [27] (Figure 2). FLC, SVP, SOC1, AP1, and SEP groups were formed, and MdFLC1c and MdFLC3 were included in the FLC group.

### 2.2. Expression Analysis of MdFLC in the Adult Trees

**Figure 3:**
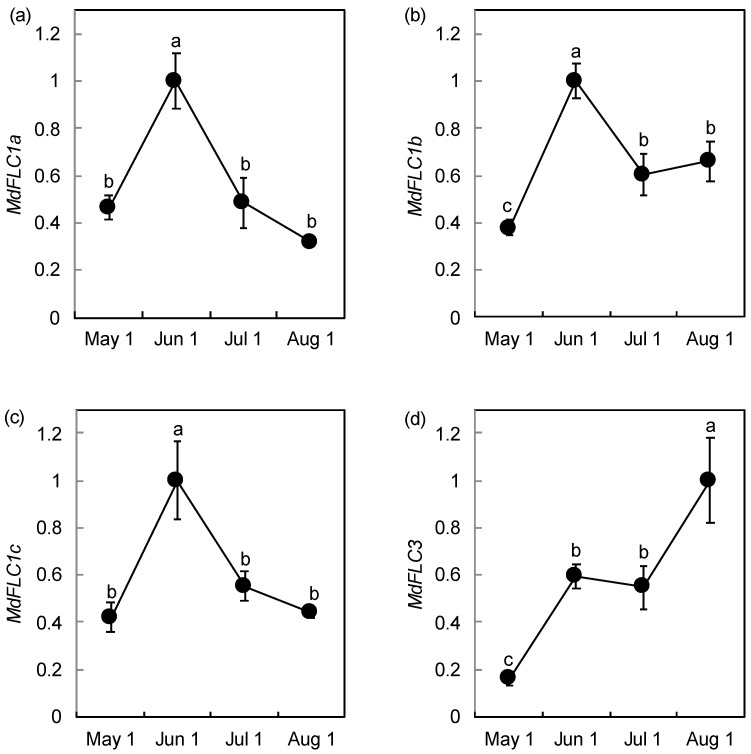
Seasonal changes in the expression levels of *MdFLC1a* (**a**), *MdFLC1b* (**b**), *MdFLC1c* (**c**), and *MdFLC3* (**d**) in the leaves of adult trees. Total RNA was prepared for the expression analysis of *MdFLC1* and *MdFLC3* on May 1, June 1, July 1, and August 1, 2016. Relative expression was determined using triplicate measurements taken from three independent biological replicates. The relative expression levels were normalized against *MdACTIN* with standard errors, and the maximum level of the transcripts was set at 1.0. The values with different letters for each gene significantly differed between days at *p* < 0.05, according to a Tukey test.

Seasonal changes in the expression of *MdFLC* were examined in the adult trees. Flowering induction occurs between late June and mid-July [27]. The expression levels of *MdFLC1a*, *MdFLC1b*, and *MdFLC1c* were high in early June and decreased in early July during the period of flowering induction (Figure 3a–c). On the other hand, the expression level of *MdFLC3* did not change from early June to early July, but increased in August (Figure 3d). FLC suppresses the expression of *FT* in leaves as mentioned above [17]. *FT* generally produces mobile floral signals in leaves [28], and FT signal movement is also reported in Rosaceae fruit trees [29]. Therefore, leaves were used for analysis in the present study.

### 2.3. Expression Analysis of MdFLC During Phase Transition

**Figure 4:**
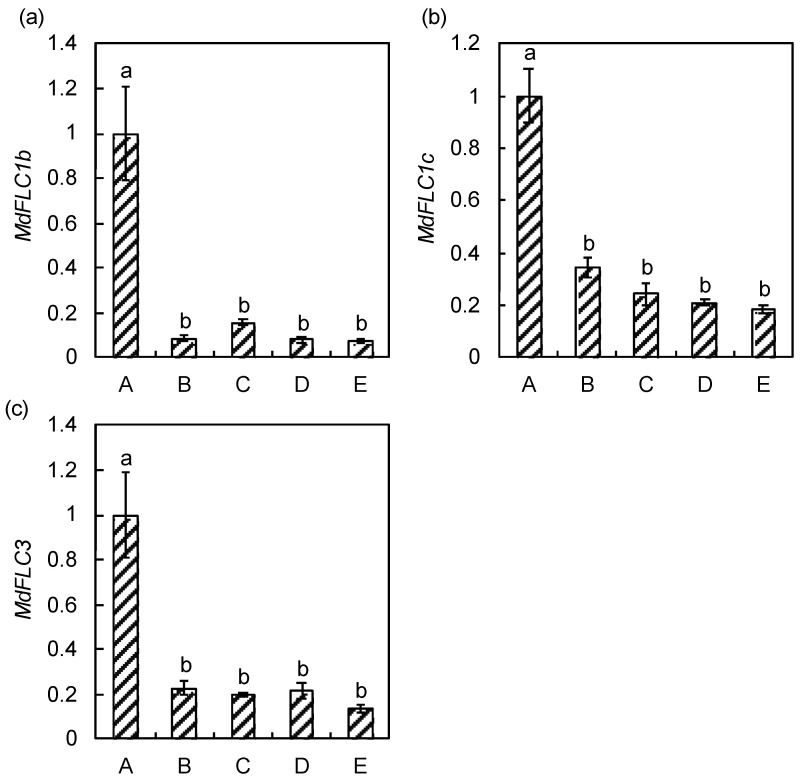
Changes in the expression levels of *MdFLC1b* (**a**), *MdFLC1c* (**b**), and *MdFLC3* (**c**) during phase transition in apple seedlings. The expression level of *MdFLC1a* was undetectable. Total RNA was prepared from the juvenile phase (A), transition phase (B and C), and adult phase (D and E) in early July 2009 as described in [27]. Relative expression was determined in triplicate measurements taken from three independent biological replicates. The relative expression levels were normalized against *MdACTIN* with standard errors, and the maximum level of the transcripts was set at 1.0. The values with different letters for each gene significantly differed between positions at *p* < 0.05 according to a Tukey test.

The expression level of *MdFLC* during phase transition was performed in apple seedlings. While *MdFLC1a* expression was not detectable, the expression levels of *MdFLC1b* and *MdFLC1c* were high during the juvenile phase and low during the transitional and adult phases (Figure 4). The expression level of *MdFLC3* during the juvenile phase was also high compared to that in the transitional and adult phases, and it was 7.4 times the level in the adult phase.

### 2.4. Transformation of Arabidopsis with MdFLC3 cDNA

**Figure 5:**
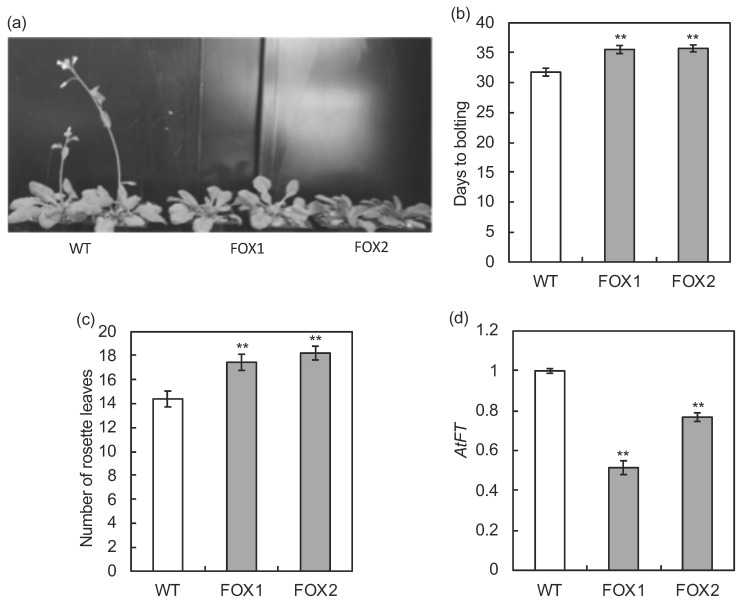
The overexpression of *MdFLC3* and phenotypic analysis in *Arabidopsis*. Flowering phenotypes 32 days after sowing (**a**), days to bolting from sowing (**b**), the number of rosette leaves at bolting time (**c**), and the expression levels of *AtFT* (**d**) in the *MdFLC3* transgenic (FOX1, 2) and wild-type (WT) plants grown under a 16 h photoperiod. The values with ** significantly differed between FOX and WT plants at *p* < 0.01, according to the Dunnett test. The values in (**b**,**c**) indicate means and standard errors (n = 30 or 31). The relative expression of *AtFT* was determined from triplicate measurements of three independent biological replicates 15 days after sowing (**d**). The relative expression levels were normalized against *AtACTIN* with standard errors and the maximum level of the transcripts was set at 1.0.

Delays in bolting were observed in more than three transgenic plants from individual seeds (T_1_) obtained by *Agrobacterium* in planta vacuum infiltration transformation. Detailed analysis was performed on their progenies, lines FOX1 and FOX2. The growth of FOX1 and FOX2 lines was observed and expression analysis of *AtFT* was then performed (Figure 5; Figure S1a,b, Supplementary Materials). The number of days from sowing to bolting was 31.8 in the wild-type (WT) control, and that in FOX1 and FOX2 was 35.5 and 35.7 days, respectively. The number of rosette leaves at the time of bolting was 14.4 in the wild-type and 17.5 and 18.2 in FOX1 and FOX2, respectively. A lower expression level of *AtFT* was observed with late flowering in the FOX lines. A very high expression level of *MdFLC3* was confirmed in the FOX lines, whereas only a trace expression of endogenous *FLC* (*AtFLC*) was found in the FOX lines and wild-type (Figure S2, Supplementary Materials). The average value of transformants derived from individual seeds, which are different from FOX1 and FOX1 lines, at the beginning of transformation showed delayed bolting, supporting the above result (Figure S1c,d).

## 3. Discussion

A phylogenetic tree analysis, including other MADS-box genes such as SVP and SOC1, revealed that both MdFLC1 and MdFLC3 belong to the same FLC group as VvFLC [30] and PtFLC [26], which has been reported to function as a floral repressor (Figure 2). Therefore, MdFLC1 and MdFLC3 were further investigated as apple FLCs in this study. FLC is one of the MADS-box proteins, which are transcription factors having a highly conserved region of approximately 60 amino acids, MADS-box, that is involved in DNA binding and dimer formation. Many MADS-box proteins in plants are classified as MIKC-type, contain MADS-, I-, K-, and C-domains [31], and form dimers and higher multimers to function [32,33]. MdFLC1c and MdFLC3 contained these four conserved domains.

**Figure 6:**
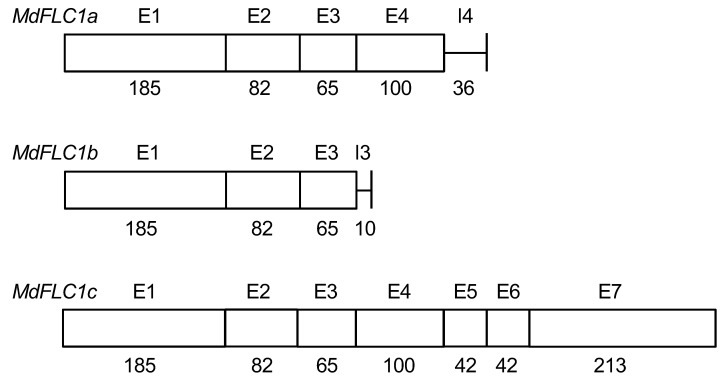
Transcript structures of *MdFLC1* splice variants, *MdFLC1a*, *MdFLC1b*, and *MdFLC1c*. E and I with numbers indicate exons and introns, respectively. Numbers under the bar correspond to their sizes, in base pairs.

Three cDNA sequences of *MdFLC1* were searched in the GDR database, and *MdFLC1* was found to correspond to MD05G1037100. *MdFLC1c* mRNA contained all exons, whereas *MdFLC1a* and *MdFLC1b* mRNA contained the sequences of the fourth and third introns, which were not removed in splicing, respectively (Figure 6). Therefore, the deduced amino acid sequence of MdFLC1c contained MADS-, I-, K-, and C-domains, whereas the sequences of MdFLC1a and MdFLC1b lacked K- and C- domains because of the stop codons in the intron sequences of their mRNA (Figure 1). K- and C-domains are important for protein–protein interactions and other functions in MIKC-type MADS proteins [34]. These results suggest that *MdFLC1c* plays the role of *MdFLC1* and that *MdFLC1a* and *MdFLC1b* are expected to be non-functional. However, since the regulation of expression by selective splicing has been reported in plant response to environmental stress [35], expression analysis was performed in the three splicing variants of *MdFLC1*. The *MdFLC3* cDNA sequence was consistent with that of MD10G1041100 in the GDR database, and its deduced amino acid sequence contained the four domains of an MIKC-type MADS protein (Figure 1), suggesting that MdFLC3 functions as an MIKC-type MADS protein.

In the present study, the expression levels of *MdFLC1a*, *MdFLC1b*, and *MdFLC1c* decreased during the period of flowering induction in a seasonal expression pattern in the adult trees. In the annual growth cycle of apple, *MdFT1*, which is a floral integrator, shows high expression, and the expression level of *MdTFL*, which is a floral repressor, decreases during the period of flowering induction [9,27]. Therefore, the expression of *MdFLC1a*, *MdFLC1b*, and particularly *MdFLC1c*, which is expected to translate functional proteins, is likely involved in suppression of flowering in the annual growth cycle of adult apple trees, as is *MdTFL*. In contrast, the expression level of *MdFLC3* did not decrease during the period of flowering induction, suggesting that *MdFLC3* is not involved in flowering induction in the annual growth cycle of adult trees.

Because phase change is observable in individuals, seedlings can be used for expression analysis of phase transition [36]. Five sites in the seedlings used in this study show phase transition based on juvenile characteristics such as flower bud formation, leaf size, and leaf serration, and were used for *MdFLC* expression analysis [27]. Although expression of *MdFLC1a* was not detected, the expression levels of *MdFLC1b* and *MdFLC1c* were high in the juvenile phase and low in the transitional and adult phases. The expression level of *MdFLC3* was similarly high in the juvenile phase. The expression pattern of *MdFLC3* suggests that it does not play a role in flowering in the annual growth cycle but acts specifically in juvenility. A similar seasonal expression pattern was observed in *MdFLC3* within buds in the adult trees and confirmed the specific role of MdFLC3 (Figure S3, Supplementary Materials). Furthermore, the expression of *MdFLC1c* in the juvenile phase of seedlings is significantly lower than the expression in the adult trees (approximately 1/6, data not shown). Therefore, we focused on this specific role of *MdFLC3* and subjected *MdFLC3* to functional analysis by the transformation of *Arabidopsis*. The results showed that *MdFLC3* is a floral repressor, confirming its role in the juvenility of apple. Since the result for phase transition was obtained in crossed seedlings, a genotype-specific effect could not be excluded. Further study using other several genotype combinations and apomixis will be necessary to confirm the findings of the present study.

FLC suppresses the expression of *FT* containing the CArG box, which is an FLC-binding sequence, in the promoter region and first intron in *Arabidopsis* leaves [17]. Apples also have sufficient genomic information and such binding sites can be analyzed. Since the *MdFTs* in the database have CArG box-like sequences in the intron and promoter region (Table 1), it can be expected that MdFLC1 and MdFLC3 bind to these sequences to control annual flowering and juvenility. Regarding the molecular mechanism of juvenility of woody plants, including fruit trees, little is known about genes located upstream of the flowering pathway. Our results suggest that the apple homolog of *FLC*, which appears to play a central role relatively upstream of the flowering pathway, could be involved in juvenility as well as in annual flowering. Testing the correlation between MdFLC characteristics and the length of the juvenile phase could provide valuable insight into the function of MdFLC in the regulation of this process in the near future. In addition, if this gene could be used as a marker, it would be possible to breed cultivars with a short juvenile period as well as various useful traits by marker-assisted selection [21,37]. As apples have advanced in genome information and genome editing technology [38], accumulating results as a model will be utilized for other fruit trees.

## 4. Materials and Methods

### 4.1. Plant Materials

Mature leaves in the juvenile phase of 8-year-old apple (*Malus domestica*) seedlings of a cross between ‘Fuji’ and ‘Himekami’ were used for cDNA isolation and sequence analysis of *MdFLC*. Mature leaves from 11-year-old apple trees (‘Fuji’ grafted onto M.9, *M. prunifolia*) were used as adult trees. For expression analysis in adult trees, mature leaves were sampled from 20-year-old apple trees (‘Fuji’ grafted onto M.9, *M. prunifolia*) on May 1, June 1, July 1, and August 1, 2016. For expression analysis of phase transition, mature leaves were collected from the juvenile to adult phase in 8-year-old apple seedlings of a cross between ‘Fuji’ and ‘Himekami’ [27]. All samples were collected in the Tohoku University (Sendai, Japan) experimental field at 38°16′ N and 140°52′ E.

### 4.2. cDNA Isolation, Sequence Analysis, and Phylogenetic Tree Analysis of MdFLC

To isolate *MdFLC1* cDNA, the apple EST sequences were searched using DDBJ and GDR. DDBJ was also used to search homologous genes in other plants. The cetyltrimethylammonium bromide (CTAB) method [27] and a TaKaRa RNA PCR kit (AMV) (Takara-Bio, Kusatsu, Japan) were used for RNA extraction and reverse transcription, respectively. Cloning of *MdFLC3* cDNA was performed by PCR with degenerate primers MdMADSF and MdMADSR (Table S1, Supplementary Materials) based on highly conserved sequences in the MADS-box protein using PROSITE (https://prosite.expasy.org). The restriction sites of *Eco*RI and *Bam*HI were added to the primers in advance. PCR was performed with cDNA from leaves in the juvenile and adult phases. PCR products were electrophoresed on agarose gel, and amplified fragments of the expected size were collected using TaKaRa RECOCHIP (TaKaRa). pUC18 was digested with *Eco*RI and *Bam*HI, and the PCR fragments were ligated into this vector and then transformed into *Escherichia coli*. The plasmid was purified for sequence analysis. Sequence Alignment by ClustalW (http://align.genome.jp) was used to prepare the sequence alignments and phylogenetic trees.

### 4.3. Expression Analysis by Real-Time PCR

For expression analysis in the adult trees, RNA extraction and reverse transcription were performed using a Cica Geneus RNA prep kit for Plant (Kanto Kagaku, Tokyo, Japan) and ReverTra Ase qPCR RT Master Mix with gDNA Remover (TOYOBO, Osaka, Japan), respectively. THUNDERBIRD SYBR qPCR Mix (TOYOBO) was used for the subsequent PCR. For expression analysis with phase transition, RNA extraction and reverse transcription were performed using the CTAB method [27] and QuantiTect Reverse Transcription Kit (Qiagen, Hilden, Germany), respectively. A QuantiTect SYBR Green PCR Kit (Qiagen) was used for the subsequent PCR. Real-time PCR was performed as described in Ikeda et al. [39], using the primer sets listed in Table S1. Coefficients of variant for quantification cycle of the reference genes among samples were 1.85%, 2.75%, and 1.01% in Figure 3, Figure 4 and Figure 5, respectively.

### 4.4. Transformation of Arabidopsis with MdFLC3 cDNA

The translated region of *MdFLC3* cDNA was amplified by PCR using Q5 DNA polymerase (New England Biolabs, Ipswich,, MA, USA) electrophoresed in an agarose gel. It was then collected from the gel and used as an insert. The primers used were MdFLC3InsertF and MdFLC3InsertR, in which *Bam*HI and *Sac*I sites were added to the 5ʹ and 3ʹ ends, respectively (Table S2, Supplementary Materials). The *GUS* sequence in the binary vector pBI121 was excised using *Bam*HI and *Sac*I, and the *MdFLC3* insert was introduced into the vector instead. The resultant vector containing *MdFLC3* cDNA with CaMV *35S* promoter was used for the transformation of *Arabidopsis thaliana* ecotype Columbia (Inplanta Innovation Inc., Yokohama, Japan). Transformed seeds (T_1_) were selected in a medium containing kanamycin, and a transgene check was performed by PCR using the primer set for *MdFLC3* (Table S2). Expression analysis was performed by real-time PCR using the primer sets for *AtFT*, *MdFLC3*, and *AtFLC*, and the *Arabidopsis* actin primer was set as a reference (Table S2). Seeds (T_2_) were collected separately from individuals (T_1_) derived from individual seeds initially obtained by *Agrobacterium* in planta vacuum infiltration transformation and each of them was sown as a line. In the T_2_ generation, segregation of the transgene was checked in each line and seeds (T_3_) were collected. Homozygous seeds that did not segregate in T_3_ were used. The homozygous seeds (T_3_) were planted and grown in vermiculite/pearlite (1:1) at 22 °C under a 16 h photoperiod, and the number of rosette leaves and days after sowing was measured at bolting [40].

## Figures and Tables

**Table 1:** Putative CArG boxes in *MdFT* ^a^.

Gene	Accession Number	Putative CArG Box	Strand	Position of 1st C from ATG ^b^
*MdFT1*	AB458506	AACT**CC**ATTAATT**G**CAGG	Top	+289
		TACT**CC**TTATTTT**G**TCAA	Top	+846
*MdFT2*	AB458504	CTAA**CC**ATTAATT**G**TGTT	Top	+1001
		AGAT**CC**TAAAAAA**G**TATA	Bottom	+994
		GTAT**CC**AAATAA**G**TTGC	Bottom	−159
		GTCT**CC**TAATTT**G**TTGT	Bottom	−745

^a^ The boxes in the introns and promoter regions, the upstream 1500-bp *MdFT* promoter sequences of the start codon were checked in both strands according to Helliwell et al. [17]. ^b^ The boxes in the introns and the promoter regions are shown with the positive and negative number of positions, respectively.

## Supplementary Materials

Supplementary materials can be found at https://www.mdpi.com/1422-0067/21/12/4562/s1. Table S1. Primers used for cloning of cDNA encoding *MdFLC* and real-time PCR in apple; Table S2. Primers used for transformation of *Arabidopsis* with *MdFLC3* cDNA and real-time PCR in transgenic *Arabidopsis*; Figure S1. Phenotype analysis in the *MdFLC3* transgenic (FOX) and wild-type (WT) plants as support data for Figure 5; Figure S2. Expression levels of *MdFLC3* and *AtFLC* in the *MdFLC3* transgenic (FOX1, 2) and wild-type (WT) plants; Figure S3. Seasonal changes in the expression levels of *MdFLC3* in the buds of adult trees.

## Abbreviations

FLC	FLOWERING LOCUS C
TFL1	*TERMINAL FLOWER 1*
FT	FLOWERING LOCUS T
LFY	LEAFY
AP1	APETALA1
EST	expressed sequence tag
DDBJ	DNA Data Bank of Japan
WT	wild-type
