# Supplementary material for: Characterization of FLOWERING LOCUS C Homologs in Apple as a Model for Fruit Trees
Source: Int J Mol Sci. 2020 Jun 26;21(12):4562. doi: 10.3390/ijms21124562 (PMC7348945; doi:10.3390/ijms21124562)
Supplement: Supplementary file 1 [file ijms-21-04562-s001.pdf]

Table S1. Primers used for cloning of cDNA encoding *MdFLC* and real-time PCR in apple.

Primer	Sequence
MdMADSF	CGGAATTCATGGNMGNGGNAARRT
MdMADSR	CGGGATCCACYTCNGCRTCRCANA
MdFLC1aF	CTCTGAAAATGATTCAAAGTGATATG
MdFLC1aR	GCAACCTTTCTTGATCTTGTTC
MdFLC1bF	CAAGGATGCTGGTGGTACTG
MdFLC1bR	CACTGCAAACACATAATCAGACC
MdFLC1cF	GACACGGACCAAACCAACTAAC
MdFLC1cR	ATCTCGGTGAATCGAATCAGTT
MdFLC3F	TGGAGCGTTACCAGATGCATAG
MdFLC3R	GGTCTTGTGCCTCCCTACCA
MdACTINF	ACTCGCATTGCTAGGGTTTC
MdACTINR	ATACCACGCTTGGACTGAGC

Table S2. Primers used for transformation of *Arabidopsis* with *MdFLC3* cDNA and real-time PCR in transgenic *Arabidopsis*.

Primer	Sequence
MdFLC3InsertF	GGATCCCCGGGTGGTCAGTCAAAAA TGGGGCGAGGGAAGGTAG
MdFLC3InsertR	GCGCCAGGAGAGTTGTTGATTTATA GTCGGAGGAAGTGCTCTGCT
AtFTF	AGAGTTGAGATTGGTGGAGAAGAC
AtFTR	CCAGTTGTAGCAGGGATATCAGTC
MdFLC3F	TGGAGCGTTACCAGATGCATAG
MdFLC3R	GGTCTTGTGCCTCCCTACCA
AtFLCF	GCCTCCGGCAAGCTCTAC
AtFLCR	TGATCCCACAAGATTATAAAG
AtACTINF	ACATTGTGCTCAGTGGTGGA
AtACTINR	GAGATCCAACATCTGCTGGAAT

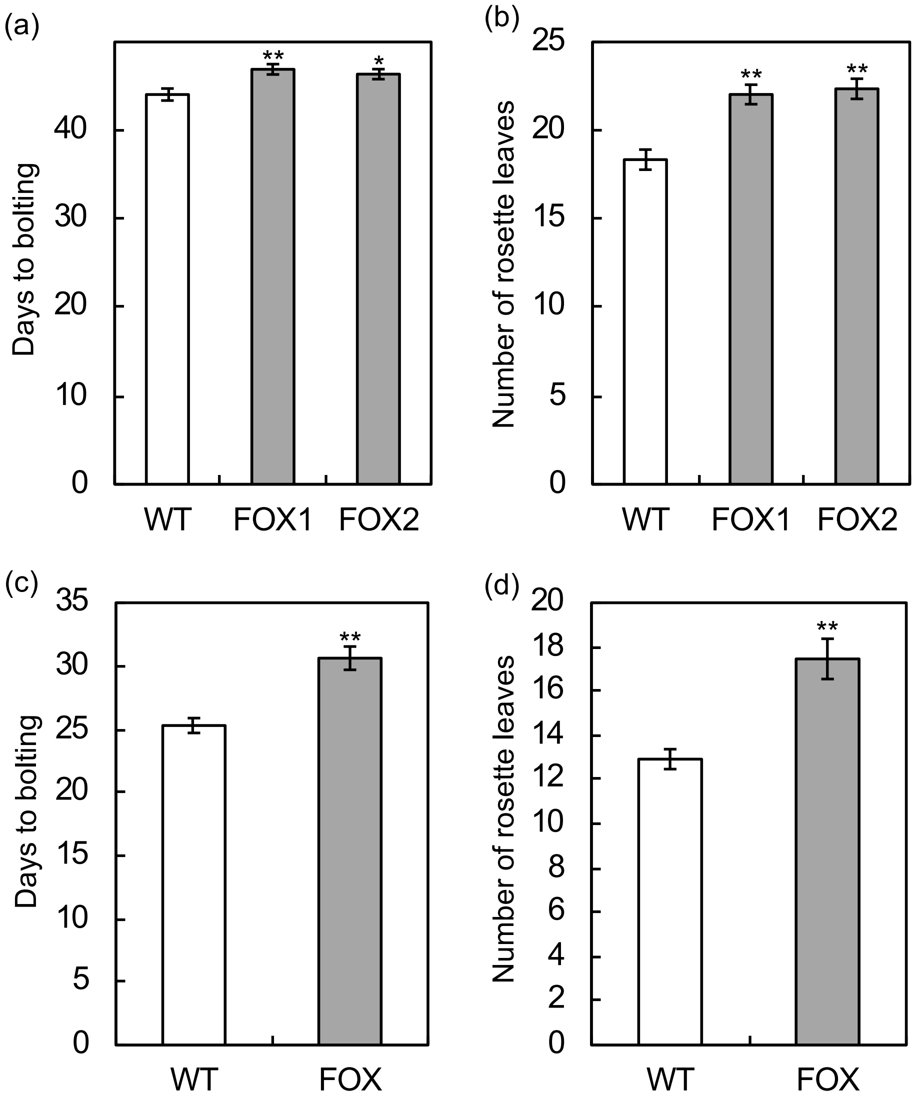

Figure S1. Phenotype analysis in the *MdFLC3* transgenic (FOX) and wild-type (WT) plants as support data for Figure 5. Days to bolting from sowing (a) and the number of rosette leaves at bolting time (b) in two FOX lines (n = 30 and 34) and WT (n = 20) plants in an experiment different from Figure 5. The values significantly differed between FOX and WT at $P < 0.01$ (**) and 0.05 (*), according to the Dunnett test. Days to bolting from sowing (c) and the number of rosette leaves at bolting time (d) in FOX (n = 13) and WT (n =14) plants. The values significantly different between FOX and WT at $P < 0.01$), according to the *t*-test.

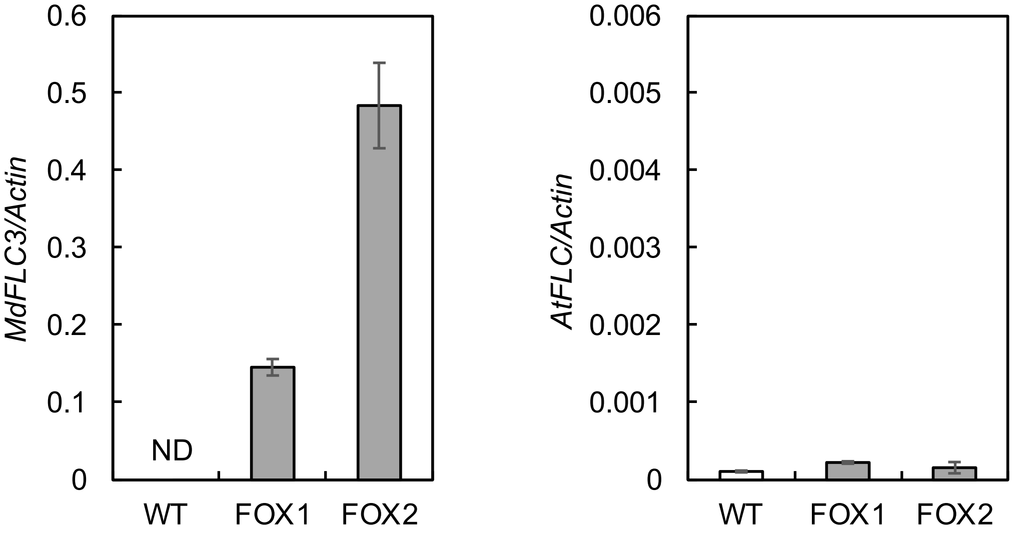

Figure S2. The expression levels of *MdFLC3* and *AtFLC* in the *MdFLC3* transgenic (FOX1, 2) and wild-type (WT) plants. The relative expression was determined from triplicate measurements of three independent biological replicates 15 days after sowing, and the relative expression levels were normalized against *AtACTIN* with standard errors. While a high expression of *MdFLC3* was observed in FOX plants, the expression of *AtFLC* was almost at the limit of detection in both FOX and wild-type plants.

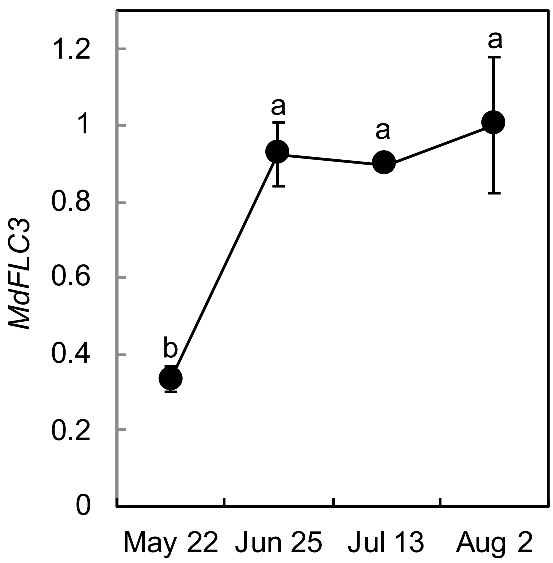

Figure S3. Seasonal changes in the expression levels of *MdFLC3* in the buds of adult trees. Total RNA was prepared for the expression analysis of *MdFLC3* on May 22, June 25, July 13, and August 2 in a different year than leaf expression in Figure 3. Relative expression was determined using triplicate measurements taken from three independent biological replicates. The relative expression levels were normalized against *GAPDH* with standard errors, and the maximum level of the transcripts was set at 1.0. The values for each gene significantly differed between days at $P < 0.05$, according to a Tukey test.
